# Generation of intestinal organoids derived from human pluripotent stem cells for drug testing

**DOI:** 10.1038/s41598-020-63151-z

**Published:** 2020-04-06

**Authors:** Shinpei Yoshida, Hiroto Miwa, Tomoyuki Kawachi, Shoen Kume, Koji Takahashi

**Affiliations:** 1 Drug Metabolism & Pharmacokinetics, Research Laboratory for Development, SHIONOGI & CO., LTD., 3-1-1, Toyonaka, 561-0825 Osaka Japan; 2 Drug Discovery Technologies, Drug Discovery & Disease Research Laboratory, SHIONOGI & CO., LTD., 3-1-1, Toyonaka, 561-0825 Osaka Japan; 3 Department of Life Science and Technology, School of Life Science and Technology, Tokyo Institute of Technology, Yokohama, 226-8501 Kanagawa Japan

**Keywords:** Pharmaceutics, Induced pluripotent stem cells

## Abstract

Drug absorption via the intestinal tissue is modulated by membrane permeability and metabolism in intestinal epithelial cells (IECs). In drug discovery research, using human IECs to evaluate membrane permeability and metabolic stability can offer very useful information when exploring for drug candidate compounds that have good bioavailability and when trying to predict the fraction absorbed and intestinal availability in humans. Here, we evaluated the pharmacokinetic functions of human IECs differentiated from human induced pluripotent stem cells (hiPSCs) in 3D cultures. As human IECs differentiated in 3D cultures form intestinal organoids and spheroids (herein termed organoids), their morphology makes it difficult to evaluate their pharmacokinetic functions. Therefore, we dissociated intestinal organoids into single cells and attempted to purify human IECs. We found that hiPSC-derived IECs (hiPSC-IECs) expressed the epithelial cell adhesion molecule (EpCAM) and could be highly purified by sorting EpCAM+ cells. The hiPSC-IEC monolayer showed a high TEER value (approximately 350 Ω × cm^2^). In addition, hiPSC-IECs oxidatively metabolized terfenadine (CYP3A and CYP2J2 substrate) and midazolam (CYP3A substrate). These results indicated that hiPSC-IECs form tight-junction and have cytochrome P450 enzymatic activities. In conclusion, we developed a novel application of hiPSC-derived intestinal organoids for drug testing.

## Introduction

To find drug candidate compounds that have good bioavailability, evaluation of their water solubility, membrane permeability and metabolic stability are important. As *in vitro* high-throughput membrane permeation screening tools for drug candidate compounds, the parallel artificial membrane permeation assay and Caco-2 cell lines derived from human colorectal adenocarcinoma have been used^1–4^. These evaluation tools are also used to predict the fraction absorbed in humans. The Caco-2 cell lines express transporters, such as P-glycoprotein (P-gp) and peptide transporter 1 (PEPT1), which they use to actively transport drug substrates. However, the expressions of cytochrome P450 enzymes in Caco-2 cell lines are poor^5^. Therefore, Caco-2 cell lines cannot be used to predict the absorption potential including intestinal metabolic stability. An alternative screening tool to predict absorption potential in humans is offered by human primary intestinal epithelial cells (human primary IECs)^6^, but there is an issue with stable accessibility.

Human induced pluripotent stem cells (hiPSCs) should be useful for drug research not only as disease-specific hiPSCs, but also as a tool for stable supply of human cells that are not readily available. Also, hiPSCs derived from genetic variants of donors can be prepared^7^. Recently, differentiation procedures into intestinal organoids from hiPSCs using 3D culture have been reported^8^. In addition, differentiation procedures into intestinal epithelial cells (IECs) from hESCs in 2D culture have been reported^9^. IECs derived from hiPSCs (hiPSC-IECs) might be more useful compared to human primary IECs because of their stable supply and potential for preparing genetic variants for cytochrome P450 enzymes and transporters.

The hiPSC-IECs differentiated in 2D cultures form monolayers and are easy to use in membrane permeation screening for test compounds. On the other hand, those differentiated in 3D cultures form organoids, which are difficult to use for drug screening due to their morphology^10^. What is not well understood is whether differences in maturity exist between hiPSC-IECs differentiated in 3D cultures and those differentiated in 2D cultures. Here, we aimed at developing a novel application of hiPSC-derived intestinal organoids for evaluation of membrane permeation and metabolism of drug candidate compounds. For this purpose, we attempted to dissociate intestinal organoids into single cells and purified hiPSC-IECs using an epithelial cell surface marker EpCAM^11,12^.

## Materials and Methods

The investigation followed the tenets of the Declaration of Helsinki promulgated and the Governmental Guidelines, and this study was conducted after getting the approval by the Ethics Committee on Human Tissue and Genome Research of Shionogi.

### Human iPS cell culture

A human iPS cell line (TkDA3–4) established as previously described^13^, was graciously provided by Dr. M Otsu (The Institute of Medical Science, The University of Tokyo, Tokyo, Japan). The hiPSCs were cultured on feeder layers of mitomycin C treated mouse embryonic fibroblasts (MEFs) in DMEM/F12 medium (Sigma-Aldrich, Saint Louis) supplemented with 100 U/mL penicillin (Thermo Fisher Scientific, Waltham), 100 µg/mL streptomycin (Thermo Fisher Scientific, Waltham), 2 mM L-glutamine (Thermo Fisher Scientific, Waltham), 0.55 mM 2-mercaptoethanol (Thermo Fisher Scientific, Waltham), 20% (v/v) Knockout Serum Replacement (Thermo Fisher Scientific, Waltham) and 5 ng/mL recombinant human basic fibroblast growth factor (ReproCELL, Yokohama). The MEFs were prepared from mouse fetal embryos on day 13.5 of gestation. To passage hiPSCs, a feeder layer of MEFs was removed by an enzymatic method with CTK dissociation solution (ReproCELL, Yokohama). The hiPSCs colonies were mechanically dissociated into small clumps and diluted to approximately one-third of the original concentration and then were seeded on a feeder layer of MEFs.

### Derivation of hiPSCs into intestinal lineages

The feeder layer of MEFs was removed by the method described above. The hiPSC colonies were gently dissociated into single cells with Accutase (Innovative Cell Technologies, San Diego) and seeded on hES-qualified Matrigel (Corning, Corning) coated plates at a density of 3 × 10^5^ cells/cm^2^. The cells were cultured in the above described hiPSC culture medium until they attained 90% confluency. Next, the cells were treated with 100 ng/mL recombinant human/mouse/rat Activin A (R&D Systems, Minneapolis) in RPMI 1640 media (Thermo Fisher Scientific, Waltham) supplemented with 100 U/mL penicillin, 100 μg/mL streptomycin and fetal bovine serum (Equitech-bio, Kerrville, day 1: 0%, day 2: 0.2% and day 3: 2%) for derivation into definitive endodermal cells (DE cells). After differentiation into DE cells, the cells were treated with 500 ng/mL recombinant human FGF4 (R&D Systems, Minneapolis) and 500 ng/mL recombinant human Wnt3A (R&D Systems, Minneapolis) in RPMI 1640 media supplemented with 100 U/mL penicillin and 100 μg/mL streptomycin for 4 days for derivation into midgut/hindgut cells. After differentiation into midgut/hindgut cells, the adhesive cells and three-dimensional spheroids were embedded in Matrigel containing 500 ng/mL recombinant human R-Spondin1 (R&D Systems, Minneapolis), 100 ng/mL recombinant human Noggin (R&D Systems, Minneapolis) and 100 ng/mL recombinant human EGF (R&D Systems, Minneapolis) on 24 well culture plates or 100 mm culture dishes. After the Matrigel had solidified, advanced DMEM/F12 (Thermo Fisher Scientific, Waltham) supplemented with 2 mM L-glutamine, 15 mM HEPES (Thermo Fisher Scientific, Waltham), 2% (v/v) B27 supplement (Thermo Fisher Scientific, Waltham), 100 U/mL penicillin, 100 μg/mL streptomycin, 2.5 μg/mL amphotericin B (infrequent) (Sigma-Aldrich, Saint Louis), 10 μM Y27632 (Wako, Tokyo, only on the first 3 days) and the growth factors described above were overlaid and replaced every 3 days for more than 28 days for derivation into intestinal lineages.

### Preparation of intestinal epithelial cell-derived hiPSCs

Intestinal organoids were incubated in ice-cold D-PBS containing 2 mM EDTA (Thermo Fisher Scientific, Waltham) and 1 mM dithiothreitol (Sigma-Aldrich, Saint Louis) for 30 min and were mechanically dissociated using a 18 G needle. The organoids were further dissociated using a 23 G needle after incubation in DMEM (Thermo Fisher Scientific, Waltham) containing 3U Dispase (Roche Applied Science, Penzberg), 1% fetal bovine serum and 10 μM Y-27632 for 20 min at 37 °C. Single cells were prepared by passing the dissociated cells through a 70 μm cell-strainer. The single cells were treated with human Fc receptors blocking reagent (BioLegend, San Diego or Miltenyi Biotec, Bergisch Gladbach) for 15 min at 4 °C and incubated with human EpCAM antibody-coated microbeads (Miltenyi Biotec, Bergisch Gladbach) for 15 min at 4 °C. The sample was subjected to a magnetic-activated cell sorting system (MACS) to purify hiPSC-IECs.

### HiPSC-IEC culture

The hiPSC-IECs were seeded on laminin511-E8 (Nippi, Tokyo) coated plates or Transwell inserts (Corning, Corning) at a high density of 2–3 × 10^5^ cells/cm^2^ in advanced DMEM/F12 supplemented with 2 mM L-glutamine, 15 mM HEPES, 2% B27 supplement, 1% (v/v) N2 supplement (Thermo Fisher Scientific, Waltham), 100 U/mL penicillin, 100 μg/mL streptomycin, 1 mM N-acetylcysteine (Sigma-Aldrich, Saint Louis), 2.5 μg/mL amphotericin B (infrequent) (Sigma-Aldrich, Saint Louis), 10 nM human gastrin Ι (Peptide Institute, Osaka), 500 nM A-83-01 (Tocris Bioscience, Minneapolis), 10 μM Y27632 (only on the day of seeding) and 500 ng/mL recombinant human R-Spondin1, 100 ng/mL recombinant human Noggin and 100 ng/mL recombinant human EGF. For long-term culture, the hiPSC-IECs were embedded in Matrigel at 5,000-10,000 cells on 24-well culture plates or 100-mm culture dishes. After the Matrigel had solidified, the culture medium described above was overlaid and replaced every 3 or 4 days.

### Gene expression analysis

Total RNA was extracted from hiPSCs and derivative cells from hiPSCs using the RNeasy Mini Kit (QIAGEN, Hilden) or PureLink RNA Mini Kit (Thermo Fisher Scientific, Waltham). Total RNAs of human adult small intestines pooled from five donors were purchased from Clontech (Mountain View). Reverse transcript reaction was performed using SuperScript III First-strand Synthesis SuperMix (Thermo Fisher Scientific, Waltham) to synthesize cDNA. Real-time polymerase chain reaction (PCR) was performed with Power SYBR Green PCR Mater Mix or Taqman Gene Expression Master Mix (Thermo Fisher Scientific, Waltham) or One Step TB Green PrimeScript PLUS RT-PCR Kit (TAKARA BIO, Shiga) using the Applied Biosystems StepOne Plus real-time PCR systems or the Applied Biosystems 7900HT Fast Real Time PCR System or Applied Biosystems 7500 Real Time PCR System. The mRNA expression levels were normalized relative to that of the housekeeping gene glyceraldehyde-3-phosphate dehydrogenase (GAPDH). Applied Biosystems inventoried primers and Taqman probes for CYP3A4 (assay ID: Hs00430021_m1), CYP2J2 (assay ID: Hs00356035_m1), GAPDH (assay ID: Hs02758991_g1) were purchased. Primer sequences for other genes are listed in Table 1.

**Table 1:** Primer sequences for each gene.

Gene Name	Forward sequence (5′→3′)	Reverse sequence (5′→3′)
SOX17	CTGCAGGCCAGAAGCAGTGTTA	CCCAAACTGTTCAAGTGGCAGA
FOXA2	GGTGTACTCCCGGCCCATTA	CAGAGTTAGCCGGGCCTGAA
CDX2	TTCACTACAGTCGCTACATCACCA	CTGCGGTTCTGAAACCAGATT
VILLIN (VIL1)	GCTTGGCAACTCTAGGGACTGG	TGAGGTTGCTGTTAGCATTGAACAC
MUC2	CAACCAGCACGTCATCCTGAA	GATGCAAATGCTGGCATCAAAG
LYSOZYME (LYZ)	CCGTGATCCACAAGGCATTA	GAGTTACACTCCACAACCTTGAACA
LGR5	ATGCTGGAATGTTTCAGGCTCA	CAGCCATCAAGCAGGTGTTCA
EPHB2	CTGCAGGGCCAGGAATTTG	CACCCTGTGGTTGTCCAGTGTTA
OCCULUDIN (OCLN)	GACCTGAATGGGTACATGTGTGTAA	GGAATTCTCACAACACCAACTGAAG
ZO-1	CGGGACTGTTGGTATTGGCTAGA	GCTAGGCCAGGGCCATAGTAAAG
GAPDH	GCACCGTCAAGGCTGAGAAC	TGGTGAAGACGCCAGTGGA

### Immunocytochemistry

The hiPSC-derived cells were fixed in Mildform 10 N (Wako, Tokyo) for 30 min at room temperature or methanol for 15 min at 4 °C and then were washed several times in D-PBS. Fixed cells were stored at 4 °C until staining. Before immunoreaction, the fixed cells were blocked in D-PBS containing 10% normal donkey serum (Jackson ImmunoResearch, West Grove) and 0.1% Triton X-100 for 45 min at room temperature. The cells were incubated with primary antibody overnight at 4 °C, and then were incubated with secondary antibody for 1 hour at room temperature. Intestinal organoids were fixed in Mildform overnight at 4 °C, and then incubated with 10–30% sucrose solution overnight at 4 °C. Fixed intestinal organoids were embedded in OCT compound (Sakura Finetek, Tokyo) and frozen on liquid nitrogen. The freezing blocks were stored at −80 °C until cryosectioning. Thin sections (10 μm) were cut on a cryostat and placed on a slide glass for staining. Immunoreaction was performed as described above. The antibodies and dilution rates were as follows: goat anti-SOX17, 1:50 (R&D Systems, Minneapolis); goat anti-FOXA2, 1:50 (R&D Systems, Minneapolis); mouse anti-CDX2, ready to use (BioGenex, San Ramon); goat anti-VILLIN, 1:50 (Santa Cruz Biotechnology, Dallas); rabbit anti-MUC2, 1:100 (Santa Cruz, Dallas); rabbit anti-Lysozyme, ready to use (Diagnostic BioSystems, Pleasanton); goat anti-EpCAM, 1:20 (R&D Systems, Minneapolis); mouse anti-vimentin, 1:50 (Santa Cruz, Dallas); mouse anti-occludin, 1:250 (Thermo Fisher Scientific, Waltham); mouse anti-ZO-1, 1:100 (Thermo Fisher Scientific, Waltham); mouse anti-OCCLUDIN, 1:250 (Thermo Fisher Scientific, Waltham); goat anti-EphB2, 1:40 (R&D Systems, Minneapolis); donkey anti-goat-Alexa Fluor 488, 1:500 (Thermo Fisher Scientific, Waltham); donkey anti-rabbit-Alexa Fluor 488, 1:500 (Thermo Fisher Scientific, Waltham); donkey anti-mouse-Alexa Fluor 488, 1:500 (Thermo Fisher Scientific, Waltham); donkey anti-mouse-Alexa Fluor 555, 1:500 (Thermo Fisher Scientific, Waltham). Nuclei were stained with Hoechst33342 (Thermo Fisher Scientific, Waltham). The stained cells were observed using a BZ-9000 microscope (Keyence, Osaka).

### Determination of cytochrome P450 enzyme activity

For evaluation of cytochrome P450 enzyme activity in hiPSC-IECs, terfenadine (Sigma-Aldrich, Saint Louis), a representative CYP3A and CYP2J2 enzyme substrate, or midazolam (Wako, Tokyo), a representative CYP3A enzyme substrate, at the concentration of 1 μM in Krebs-Henseleit buffer (pH 7.4) was incubated with the pre-separation cells before MACS, hiPSC-IECs or the negative fraction after MACS for 90 min at 37 °C. For evaluation of cytochrome P450 enzyme activity in hiPSC-IECs after 8-weeks culture, terfenadine at the concentration of 1 μM in Krebs-Henseleit buffer (pH 7.4), or midazolam at the concentration of 20 μM in Williams medium E (Thermo Fisher Scientific, Waltham) supplemented with 2 mM L-glutamine was incubated with hiPSC-IECs at 37 °C for 90 or 120 min, respectively. In the CYP3A enzyme activity assay using midazolam, 10 μM ketoconazole (Sigma-Aldrich, Saint Louis), a representative CYP3A enzyme inhibitor, was also added. Single cell suspension at 1 or 2 × 10^6^ cells/mL and the reaction volume of the cell suspension was 30 μL. The reactions were stopped by adding four times the volume of acetonitrile. Metabolites of terfenadine (hydroxyl-terfenadine, Sigma-Aldrich, Saint Louis) or midazolam (hydroxyl-midazolam, Sigma-Aldrich, Saint Louis) were measured using LC/MS/MS. The LC/MS/MS systems consisted of a Waters ACQUITY UPLC (Waters Corporation, Milford) and a Waters Quattro Ultima mass spectrometer (Waters Corporation, Milford) or Nexera UHPLC (Shimadzu, Kyoto) and Triple Quad 6500 Plus system (AB SCIEX, Tokyo) or Triple Quad 6500 system (AB SCIEX, Tokyo). The multiple reaction monitoring mode was used to monitor ions. The hydroxyl-terfenadine precursor ion was formed using a cone voltage of 40 V. The product ion that formed at the collision energy of 30 eV (m/z 488.30 → 452.20) was monitored. The hydroxyl-midazolam precursor ion was formed using a cone voltage of 40 or 130 V. The product ion that formed at the collision energy of 20 eV (m/z 342.00 → 324.10) or 37 eV (m/z 341.55 → 202.79) was monitored. The columns (1.7 um ACQUITY UPLC BEH C18, 2.1 × 100 mm, Waters Corporation, Milford, and CAPCELL PAK ADME, 2.1 × 50 mm, OSAKA SODA, Osaka) were used for chromatographic separation of these analytes. The mobile phases were 0.1% HCOOH in distilled water (mobile phase A) and acetonitrile (mobile phase B). The flow rate was 0.5 or 0.75 mL/min. The gradient conditions were 10-95-95-10-10 (% of B concentration) / 0-1-1.2-1.21-1.5 (min) or 30-40-40-95-95-30 (% of B concentration) / 0-0.4-0.9-0.91-1.1-1.11 or 0-0.4-0.7-0.71-0.9-0.91 (min). Standard curves were prepared in the respective cell lysate matrices.

### Assessment of monolayer integrity in hiPSC-IECs

The monolayer integrity in hiPSC-IECs was evaluated by trans-epithelial electrical resistance (TEER) and the permeability of [^14^C]-inulin and lucifer yellow (hydrophilic paracellular transport marker). TEER values were measured using Millicell-ERS (Merck Millipore, Darmstadt). For the permeability assay of [^14^C]-inulin (American Radiolabeled Chemicals Inc., Saint Louis) or lucifer yellow (Wako, Tokyo), the apical side to basal side permeation of [^14^C]-inulin (2 μCi/mL) or lucifer yellow (100 μg/mL) for 120 min incubation at 37 °C was evaluated. The culture medium was removed, and the hiPSC-IEC monolayer was rinsed twice with transport medium (Hank’s balanced salt solution, pH 7.4). [^14^C]-Inulin or lucifer yellow donor solution, 250 μL, was added to the apical side and 900 or 700 μL of transport medium was added to the basal side. The hiPSC-IEC monolayer was incubated on a shaker for 120 min at 37 °C. Subsequently, the transport medium at the basal side was sampled and the radioactivity was determined by Tri-Carb 3100 (PerkinElmer, Waltham). The fluorescence of lucifer yellow was determined by a multi-plate reader EnSpire (PerkinElmer, Waltham). The apparent permeability coefficient (Papp) was calculated for cellular transport in the hiPSC-IEC monolayer according to following equation:

1 $$Papp=\frac{dQ}{dt}\times \frac{1}{A\times C0}$$

where *dQ*/*dt*, *A*, C_0_ represent [^14^C]-inulin or lucifer yellow transported to the basal side per unit time, the surface area of the transport membrane, the initial concentration of [^14^C]-inulin or lucifer yellow on the apical side, respectively.

### Data analysis

The data are expressed as means ± S.D. of at least three independent measurements. Statistical significance was determined by two-tailed unpaired Student’s *t*-test and the difference (p value) <0.05 was considered significant.

## Results

### Differentiation of hiPSCs into intestinal lineages via definitive endoderm and midgut/hindgut cells

**Figure 1:**
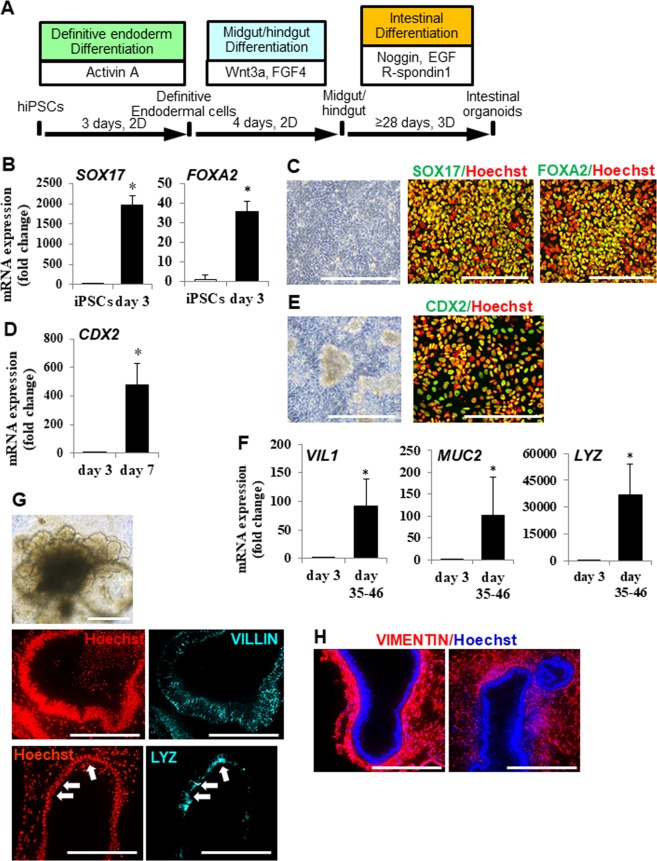
Derivation of hiPSCs into intestinal organoids. (**A**) Schematic drawing of the procedure for differentiating hiPSCs into intestinal organoids via definitive endodermal cells and midgut/hindgut cells. (**B**) Definitive endodermal cell marker (*SOX17*and *FOXA2*) gene expressions in differentiated cells at day 3. Data are presented as means ± S.D. Fold changes versus that of iPSCs are shown (hiPSCs: n = 8, differentiated cells: n = 7). ^*^p < 0.01. (**C**) Bright field image and immuno-staining images of SOX17 and FOXA2 in differentiated cells at day 3. Scale bars represent 200 µm. (**D**) Midgut/hindgut cell marker *CDX2* gene expression in differentiated cells at day 7. Data are presented as means ± S.D. Fold changes versus that of day 3 definitive endodermal cells are shown (differentiated cells at day 3: n = 7, differentiated cells: n = 7). ^*^p < 0.01. (**E**) Bright field image and immuno-staining image of CDX2 of the differentiated cells at day 7. Scale bars represent 200 um. (**F**) Enterocyte marker *VILLIN* (*VIL1*) gene, goblet cell marker *MUCIN* (*MUC2*) gene, Paneth cell marker *lysozyme* (*LYZ*) gene expressions in differentiated cells at day 35–46. Data are presented as means ± S.D. Fold changes versus that of day 3 definitive endodermal cells are shown (differentiated cells at day 3: n = 7, differentiated cells: n = 19). (**G**) Representative bright field image and immuno-staining images of VILLIN and LYZ in the differentiated into intestinal lineages are presented. The scale bars of bright field and immune-staining images represent 500 and 200 μm, respectively. Arrows depict the corresponding regions positively stained with anti-LYZ antibody in both regions. (**H**) Immuno-staining images of a mesenchymal cell marker VIMENTIN in intestinal organoids. The scale bar represents 200 μm.

We tried to differentiate hiPSCs into intestinal lineages using a 3D culture procedure^8^. The procedure for differentiation into intestinal lineages is shown (Fig. 1A). First, we examined the differentiation of hiPSCs into definitive endodermal cells. At 3 days post-differentiation, the expressions of definitive endodermal cell marker (*SOX17* and *FOXA2*) genes were up-regulated, according to real time PCR analysis (Fig. 1B). Immuno-cytochemical analysis revealed that a high proportion of differentiated cells expressed SOX17 and FOXA2 (Fig. 1C). These data indicated that hiPSCs were efficiently differentiated into definitive endodermal cells by Activin A treatment. We then examined the differentiation of definitive endodermal cells into midgut/hindgut cells. At 7 days post-differentiation, the expression of a midgut/hindgut cell marker gene, *CDX2* (Fig. 1D), but not a foregut marker gene, *SOX2* (SY *et al*., unpublished), was observed by real time PCR. Most of the differentiated cells expressed CDX2, and 3D aggregated spheroids were formed spontaneously (Fig. 1E). These data indicated that definitive endodermal cells were efficiently differentiated into midgut/hindgut cells by high concentrations of Wnt3a and FGF4. We next examined the differentiation of midgut/hindgut cells into intestinal lineages. Adherent cells on the culture plate and aggregated spheroids were scrapped off and embedded into Matrigel. At over 35 days post-differentiation, organoids and spheroids (herein termed organoids) were observed (Fig. 1G) and the expressions of an enterocyte marker gene, *VILLIN (VIL1)*, a goblet cell marker gene, *MUC2*, and a Paneth cell marker gene, *LYSOZYME* (*LYZ*) were observed (Fig. 1F). The cells in organoids expressed VILLIN and LYZ (Fig. 1G). These data indicated that midgut/hindgut cells differentiated into intestinal lineages. Moreover, the surrounding cells of organoids expressed a mesenchyme cell marker VIMENTIN (Fig. 1H).

### Purification of hiPSC-IECs from intestinal organoids

**Figure 2:**
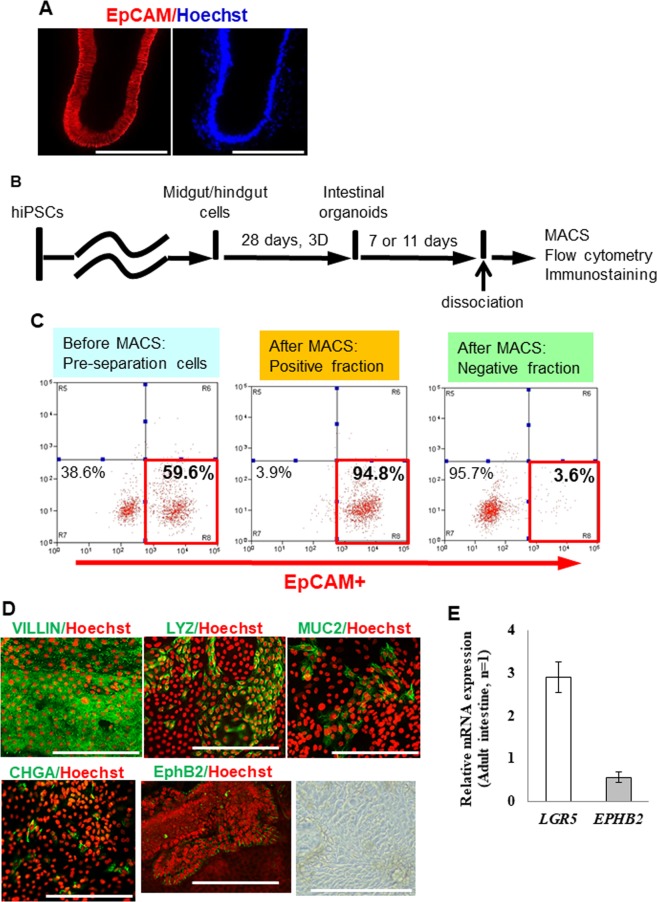
Purification of hiPSC-IECs from intestinal organoids. (**A**) The immuno-staining images of EpCAM in intestinal organoids are shown. The scale bar represents 200 μm. (**B**) A schematic drawing of the assay procedure. (**C**) The proportion of EpCAM^+^ cells in pre-separation cells before MACS, positive fraction after MACS and negative fraction after MACS are analyzed by flow cytometry. (**D**) Representative immuno-staining images of VILLIN, LYZ, MUC2, CHGA, and EphB2 in EpCAM^+^ cells (from the left), and bright field image (the rightmost panel) seeded on a culture plate. A scale bar represents 200 µm. (**E**) Intestinal stem cell marker (*LGR5*) and transient amplifying cell maker (*EPHB2*) gene expressions in EpCAM^+^ cells. Data are presented as means ± S.D. (n = 3).

Because intestinal organoids derived from hiPSCs contained iPSC-IECs and mesenchymal cells (Fig. 1H), we attempted to purify hiPSC-IECs. We focused on an epithelial cell adhesion molecule EpCAM (also known as CD326), which is expressed in epithelial cells of several tissues including IECs^11^. EpCAM was expressed in hiPSC-IECs but not in mesenchymal cells (Fig. 2A). We then tried to purify hiPSC-IECs by magnetic-activating cell sorting (MACS) using human EpCAM antibody-coated microbeads. To confirm the hiPSC-IECs sorted by MACS, we examined the ratios of EpCAM^+^ cells in the pre-separation cells, the positive and negative fractions after MACS by flow cytometric analysis. The EpCAM^+^ cell ratio in the pre-separation cells, the positive fraction and the negative fraction were 59.6%, 94.8% and 3.6%, respectively (Fig. 2C). Most of the cells in the positive fraction expressed VILLIN. Goblet cells (MUC2^+^), Paneth cells (LYZ^+^) and enteroendocrine cells (CHGA^+^) were also present in the positive fraction, although these cells were at a lower proportion compared to enterocytes (Fig. 2D). We also found the existence of cells expressing the transient amplifying cell marker, EphB2 (Fig. 2D). The expression of an intestinal stem cell marker gene, *LGR5*, was observed at a level comparable to that of the adult intestine (Fig. 2E). These results indicate that hiPSC-IECs were highly enriched by MACS using human EpCAM antibody-coated microbeads.

### Evaluation of characteristic functions of hiPSC-IECs

**Figure 3:**
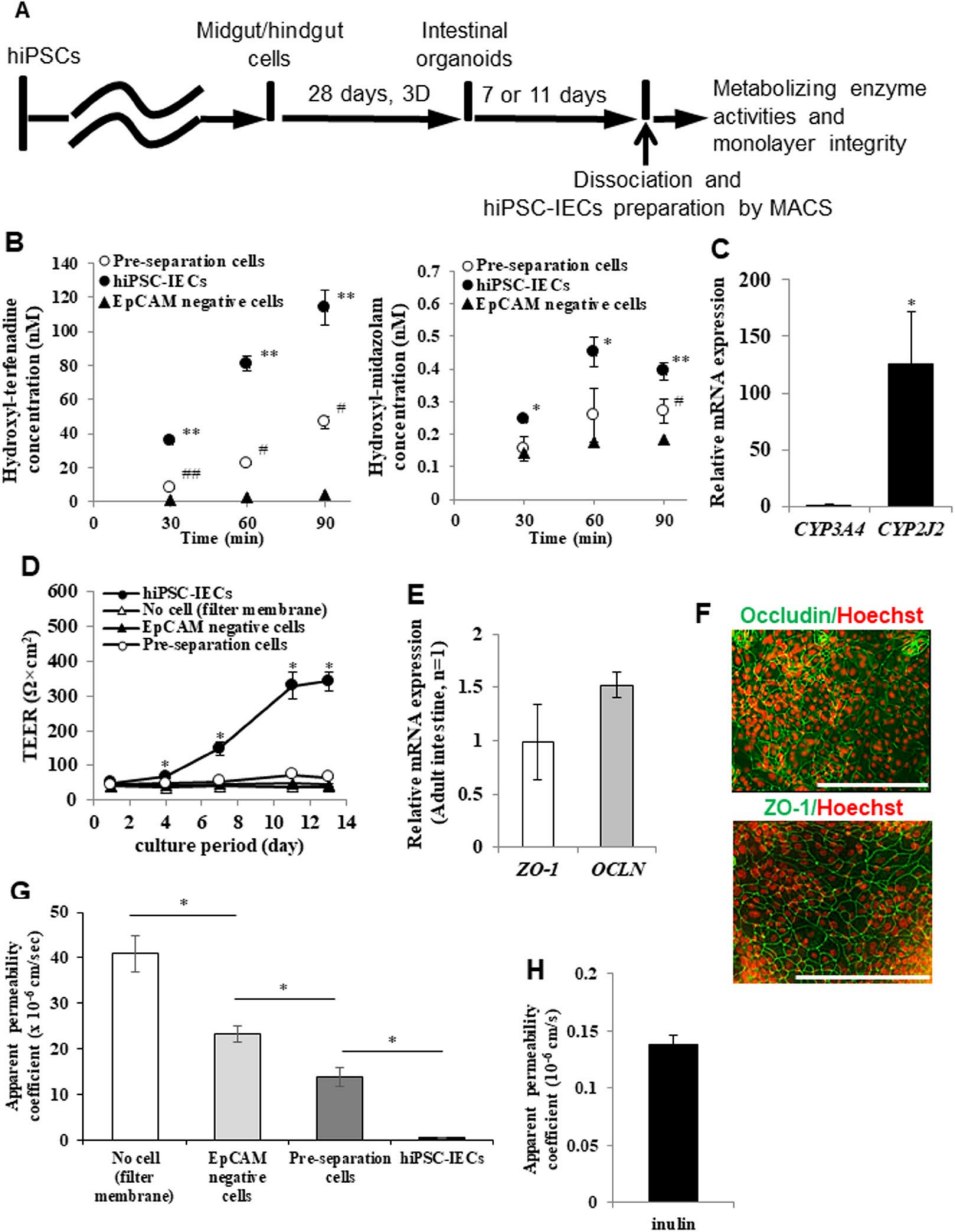
Functional characterization of hiPSC-IECs. (**A**) Schematic drawing of the assay procedure. (**B**) Drug metabolizing enzyme activity in pre-separation cells before MACS, hiPSCs-IECs and negative fraction after MACS. Data are presented as means ± S.D. (n = 3, respectively). ^*^p < 0.05, ^**^p < 0.01 versus pre-separation cells before MACS; ^#^*p* < 0.05, ^##^*p* < 0.01 versus negative fraction after MACS. (**C**) *CYP3A4* and *CYP2J2* relative gene expressions in hiPSC-IECs. Data are presented as means ± S.D. (n = 3). ^*^p < 0.05. (**D**) TEER values in the filter membrane, EpCAM negative cells, pre-separation cells, and hiPSC-IECs at each culture day after seeding onto Transwell inserts. TEER measurements were conducted in culture medium. Data are presented as means ± S.D. (n = 4). ^*^p < 0.01. (**E**) Tight-junction maker (*OCLN* and *ZO-1*) gene expressions in hiPSC-IECs (n = 3). (**F**) Representative immuno-staining images of OCCLUDIN and ZO-1 in hiPSC-IEC monolayer after seeding on a culture plate. Human iPSC-IECs for ZO-1 expression analysis differentiated under a modified protocol, in which part of recombinant proteins are replaced with small molecular compounds or excluded. A scale bar represents 200 µm. (**G**) The apparent permeability coefficient of a paracellular marker lucifer yellow, in the filter membrane without cells, EpCAM negative cells, the pre-separation cells, and hiPSC-IECs at day 13 post-seeding onto Transwell inserts. Human iPSC-IECs differentiated under a modified protocol, in which part of recombinant proteins are replaced with small molecular compounds or excluded. Data are presented as means ± S.D. (n = 4). ^*^p < 0.01 versus the pre-separation cells. (**H**) The apparent permeability coefficient of paracellular marker inulin in hiPSC-IEC monolayer after seeding on a Transwell insert. Data are presented as means ± S.D. (n = 3). Permeation assay was conducted on day 13 after seeding.

IECs play an important role in the intestinal metabolism and absorption of drugs. We next evaluated the CYP enzyme activity and barrier function of hiPSC-IECs. We then determined the CYP enzyme activity in hiPSC-IECs, by evaluating hydroxylation of terfenadine and midazolam using the pre-separation cells, hiPSC-IECs or EpCAM negative cells (Fig. 3B). The means and S.D. values of metabolizing enzyme activity for terfenadine at 90 min in the pre-separation cells, hiPSC-IECs, and the EpCAM negative cells were 0.517 ± 0.125, 1.26 ± 0.04 and 0.0463 ± 0.009 pmol/min/10^6^ cells, respectively. Human iPSC-IECs exhibited a significantly higher value compared to those of the pre-separation cells and EpCAM negative cells (*p* < 0.01). The means and S.D. values of CYP3A metabolizing enzyme activities at 90 min in the pre-separation cells, hiPSC-IECs, and the EpCAM negative cells were 3.01 ± 0.39, 4.37 ± 0.30 or 2.06 ± 0.26 fmol/min/10^6^ cells, respectively. The value in hiPSC-IECs was significantly higher compared to those of the pre-separation cells and the EpCAM negative cells (*p* < 0.01). These results revealed that the purified hiPSC-IECs exhibited CYP3A and CYP2J2 metabolizing enzyme activities, with a high activity for CYP2J2 that is responsible for the hydroxylation reaction of terfenadine (Fig. 3B). This result agreed with the much higher *CYP2J2* gene expression than that of *CYP3A4* in hiPSC-IECs (Fig. 3C). We then measured the trans-epithelial electrical resistance (TEER) (Fig. 3D) to conirm the barrier function of the hiPSC IEC monolayer. The means and S.D. values of TEER of the Transwell filter membrane without cells and those of the EpCAM negative cells, the pre-separation cells, and the hiPSC-IECs at day 13 post-seeding onto the Transwell inserts were 37.5 ± 1.6, 43.3 ± 3.4, 65.6 ± 2.8 and 342 ± 23 Ω × cm^2^, respectively. The results revealed that the TEER value of hiPSC-IECs was significantly higher compared to the others (*p* < 0.05) (Fig. 3D). We also confirmed the expression of the tight-junction marker genes, *OCLN* and *ZO-1* by real-time PCR (Fig. 3E), and OCCLUDIN and ZO-1 proteins by immunocytochemistry (Fig. 3F). We determined the apical to basal apparent permeability coefficient of a hydrophilic paracellular markers lucifer yellow and inulin (Figs. 3G and 3H). The means and S.D. values of the apparent permeability coefficient of lucifer yellow in the Transwell filter membrane without cells and those of the EpCAM negative cells, the pre-separation cells, and hiPSC-IECs at day 13 post-seeding onto the Transwell inserts were 40.9 ± 4.0, 23.3 ± 1.7, 13.8 ± 2.0 and 0.624 ± 0.020 ×10^−6^ cm/s, respectively. The results showed that the value in hiPSC-IECs was significantly lower compared to the others (*p* < 0.01) (Fig. 3G). The mean and S.D. value of the apparent permeability coefficient of inulin in the hiPSC-IEC monolayer was 0.138 ± 0.008 ×10^−6^ cm/s, which was low, in agreement with the low value previously reported^1,4^ (Fig. 3H). Taken together, these results reveal that hiPSC-IECs showed the barrier function.

### Long-term culture of hiPSC-IECs

**Figure 4:**
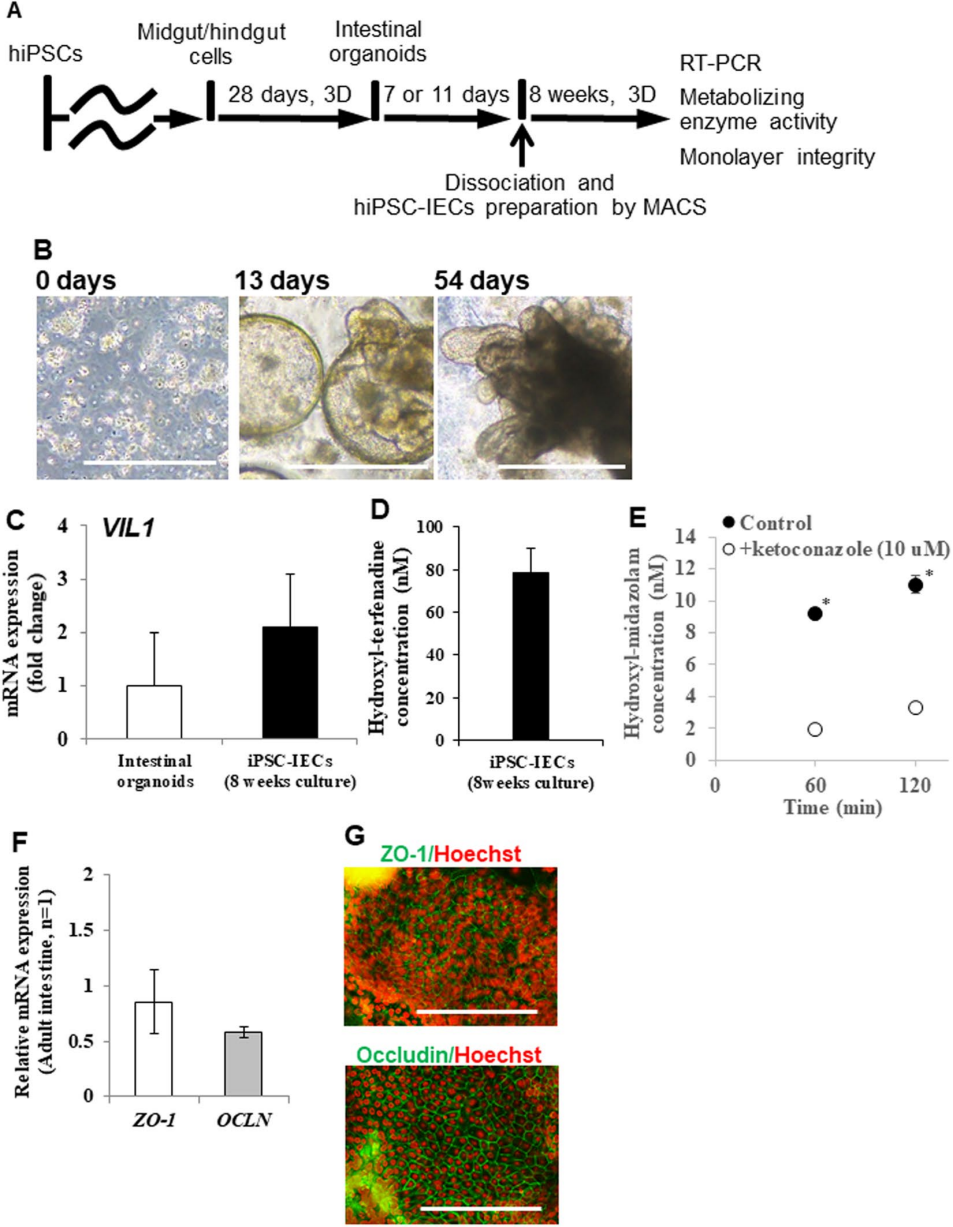
Development of long-term culture method of hiPSCs-IECs. (**A**) Schematic procedure for long-term culture of hiPSCs-IECs. (**B**) Bright field images of hiPSC-IECs on days 0, 19 and 54 are shown. Scale bars represent 500 µm. (**C**) Enterocyte marker *VILLIN* gene expression in intestinal organoids and long-term cultured hiPSC-IECs are at similar levels. Data are presented as means ± S.D. (intestinal organoids: n = 19, long-term cultured hiPSC-IECs: n = 3) (**D**) Drug metabolic activity for terfenadine in 8-weeks-cultured iPSC-IECs. Data are presented as means ± S.D. (n = 3). (**E**) Drug metabolic activity for midazolam in 8-weeks-cultured iPSC-IECs. Data are presented as means ± S.D. (n = 3). ^*^p < 0.01 versus the reaction with 10 µM ketoconazole. (**F**) Tight-junction maker (*OCLN* and *ZO-1*) gene expressions in hiPSC-IECs at day 32 post-seeding onto Transwell inserts after 8-weeks-cultured (n = 3). (**G**) Representative immuno-staining images of OCCLUDIN and ZO-1 in 8-weeks-cultured hiPSC-IEC monolayer after seeding on a culture plate. A scale bar represents 200 µm.

For a high-throughput intestinal absorption study of drug candidate compounds using hiPSC-IECs, it is not efficient to prepare hiPSCs-IECs from iPSCs for each experiment. We o establish a long-term culture method of hiPSC-IECs by following the procedure described in Fig. 4A. We tested culturing hiPSC-IECs for 8 weeks by embedding them in Matrigel supplemented with media and allowing them to form organoids and proliferate for 8 weeks (2 passages) (Fig. 4B). Subsequently, we confirmed the expression of the enterocyte marker *VILLIN* gene expression in long-term cultured hiPSC-IECs and revealed that the expression level was similar to that of intestinal organoids derived from hiPSCs (Fig. 4C). Finally, to confirm the maintenance of CYP3A and CYP2J2 enzyme activity and tight-junction function after long-term culture, we confirmed the hydroxylation of terfenadine and midazolam by the cells. The production of hydroxyl-terfenadine and hydroxyl-midazolam was observed. The generation of hydroxyl-midazolam was significantly inhibited by 10 μM ketoconazole (Fig. 4D,E). The expressions of *OCLN* and *ZO-1* genes were at levels comparable to those of the adult intestine (Fig. 3F). The expressions of OCCLUDIN and ZO-1 proteins were observed by immunocytochemistry (Fig. 3G).

## Discussion

The hiPSCs can serve as an attractive tool for drug development and studies of disease mechanisms^14^. Recently, differentiation procedures into IECs from hESCs or hiPSCs have been reported^8–10,15^. Human iPSCs possess high proliferative activity and can be differentiated into preferred cell types. Compared to primary human IECs, of which a stable supply is difficult to maintain, hiPSC-IECs could be prepared in a reproducible manner. Therefore, hiPSC-IECs should serve as a good tool for drug screening and prediction of drug absorption in humans.

We evaluated the differentiation into intestinal lineages from hiPSCs using a 3D culture procedure described previously^8^. The DE differentiation from hiPSCs was conducted using Activin A^16^, followed by midgut/hindgut differentiation from DE cells, conducted using high concentrations of both Wnt3a and FGF4, which synergistically induced midgut/hindgut differentiation^8^. The intestinal differentiation from midgut/hindgut cells was conducted using R-spondin1, noggin and EGF, which not only induced intestinal organoid differentiation from midgut/hindgut cells^8^ but also promoted proliferation of mouse and human IECs^17,18^. As a result, we confirmed differentiation into intestinal organoids from hiPSCs. However, intestinal organoids contained mesenchymal cells in addition to IECs. Mesenchymal cells might be derived from mesoderm cells that appeared during differentiation, which might be important for the development of organoids and epithelial growth^8,19^. To avoid possible effects from the mesenchymal cells on pharmacokinetic functions in hiPSC-IECs, we purified hiPSC-IECs from the intestinal organoid and replated the cells in a monolayer. As far as we know, no specific marker for IECs has yet been reported. We reveal that EpCAM, a marker for epithelial cells, is expressed in hiPSC-IECs and that human EpCAM antibody-coated microbeads are useful for purification of hiPSC-IECs. As mesenchymal cells are present in intestinal organoids derived from human pluripotent stem cells^8,19^, our purification method using EpCAM would be applicable.

Subsequently, we evaluated the pharmacokinetic functions, such as the barrier function by the tight-junction formation and drug metabolizing activities of the cytochrome P450 enzymes, of hiPSC-IECs. The hiPSC-IECs expressed OCCLUDIN and ZO-1, which are structural proteins of tight-junctions^20^. The TEER values of hiPSC-IECs monolayer were significantly higher than the filter membrane negative control, negative fraction cells, and pre-separation cells and were comparable to that of Caco-2 cells^10,21^. The apparent permeability coefficient of lucifer yellow of the hiPSC-IECs monolayer was significantly lower than the filter membrane negative control, EpCAM negative cells and pre-separation cells. The apparent permeability coefficient of inulin of the hiPSC-IECs monolayer showed a low value^1,4^. These results indicated that hiPSC-IECs have a barrier function. We evaluated CYP3A and CYP2J2 metabolizing enzyme activities, which are forms of cytochrome P450 expression in the human intestine^22^. *CYP2J2* enzyme expression was detected at a much higher level than *CYP3A4* in hiPSC-IECs. Generation of hydroxyl-midazolam and hydroxyl-terfenadine was observed, with the generation of hydroxyl-terfenadine being much higher than that of hydroxyl-midazolam. These results indicated that hiPSC-IECs exhibited both CYP3A and CYP2J2 enzymatic activities, with CYP2J2 enzymatic activity was higher than that of CYP3A, which agreed well with the mRNA expression result that *CYP3A4* was lower than that of *CYP2J2* in hiPSC-IECs (Fig. 3C). On the other hand, in human adult intestinal tissues, the protein expression of CYP3A was reported to be higher than that of CYP2J2^22^. Therefore, our findings on the expressions of CYP3A and CYP2J2 in hiPSC-IECs were different from those of human adult intestinal tissues. Because hiPSC-IECs are reported to be immature compared to adult IECs^23,24^, cytochrome P450 expressions may be associated with maturation in hiPSC-IECs. Cytoplasmic VILLIN expression was observed in hiPSC-IECs (Fig. 2D). As VILLIN is localized to the cytoplasm in early embryonic stage^25^, hiPSC-IECs might be immature. Therefore, further maturation of hiPSC-IECs would be required. Procedures that could be useful include heterotopic transplantation into a kidney capsule^24^, transplantation of intestinal grafts prepared by bioengineering technique^26^, co-culture with intestinal neurons^27^ and myocytes, suppression of the transcriptional repressor B lymphocyte-induced maturation protein 1 (Blimp1) gene^28,29,30^, and stimulation by diet ingredients.

Finally, we attempt to establish a maintenance culture procedure for a prolonged period. A long-term culture procedure is useful for drug screening^31^. When hiPSC-IECs were embedded in Matrigel supplemented with media for 8 weeks, they proliferated, formed organoids and expressed the *Villin* gene. Under our current protocol, the CYP3A and 2J2 enzymatic activities could be maintained for 8 weeks and were comparable to those of the freshly generated hiPSC-IECs (Figs. 3B and 4D,E). The OCCLUDIN and ZO-1 protein were also expressed after 8-weeks culture. However, the increase of TEER values was not observed and the permeability of lucifer yellow was high (SY unpublished data).

Therefore, a refinement of a long-term culture procedure, for example, the optimal culture period, would be preferable to perform a high-throughput intestinal absorption study of drug candidate compounds.

In this study, we revealed that hiPSC-IECs expressed barrier function by the tight-junction formation and drug metabolizing activities of the cytochrome P450 enzymes. However, additional information on phase II drug metabolizing enzyme activities and intracellular drug transporting via transporter would be valuable, and the present hiPSC-IECs system would be useful for this.

## Conclusion

We succeeded in purifying hiPSC-IECs from intestinal organoids and confirming their pharmacokinetic functions. In drug discovery research, evaluation of membrane permeability and metabolic stability using human IECs can be very useful for the exploration of drug candidate compounds that have good bioavailability and for predicting the fraction absorbed in humans. In this study, we indicated that a novel application of intestinal organoids derived from hiPSCs for drug candidate screening and prediction of bioavailability in humans.
